# Towards Sustainable Open Heart Surgery in Zimbabwe

**DOI:** 10.3389/fped.2022.806411

**Published:** 2022-07-05

**Authors:** Simukayi Percy Machawira, Wilfred Muteweye, Emmerson Mutetwa, Shield Kajese

**Affiliations:** ^1^Department of Cardiothoracic Surgery, Parirenyatwa Group of Hospitals, Harare, Zimbabwe; ^2^Department of Cardiothoracic Surgery, University of Zimbabwe, Harare, Zimbabwe; ^3^Department of Surgical Sciences, Faculty of Medicine and Health Sciences, University of Zimbabwe, Harare, Zimbabwe; ^4^Department of Anaesthetics and Critical Care Medicine, University of Zimbabwe, Harare, Zimbabwe

**Keywords:** sustainable, cardiac surgery, open heart surgery, low income country, Zimbabwe

## Abstract

Open heart surgery has become more readily available worldwide, especially in the West, whilst it remains elusive for the majority of the people in Sub-Saharan Africa, and Zimbabwe in particular. Efforts to provide the service began in the 1950s and open heart surgery became a regular service from 1989 to 2003. From 1997, Zimbabwe went through a phase of political and economic turmoil resulting in the disruption of meaningful service. This study seeks to make the case for prioritization of domestic resources toward open heart surgery, taken as learning from African countries that faced a similar predicament such as Ghana but who have since been able to sustain their programs. To ensure the success of the program, the following are necessary: the requisite personnel, equipment, consumables, and competitive remuneration. The plan is to work with Government, the private sector, and other players to harness resources toward sustainable open heart surgery in Zimbabwe.

## Introduction

Zimbabwe is a land-locked country in southern Africa with a population of 15.17 million people (1). The population is fairly young with almost 50% of the population below the age of 18 years. Zimbabwe being a developing country has a significant burden of rheumatic heart disease and this affects a significant number of children. The true disease burden is not well documented, however, data from other African countries suggest a similar disease pattern (2–5). Worldwide, the rate of birth of children with congenital heart diseases remains unchanged at roughly 0.8% per live birth (2, 6). At least 70% of congenital heart defects are simple defects that can often be cured by surgery (7). The thrust for the Government and non-governmental organizations has been primary health, however, current literature has shown that non-communicable diseases are now the major cause of mortality in low and middle-income countries (8).

Zimbabwe has gone through great economic, social, and political changes over the past 25 years, and this has affected the delivery of cardiac surgery to the greater population. The situation has worked as detrimental to the majority of the patient population as they have become poorer and disenfranchised. Cardiac diseases in children, in particular, and the whole population at large have been neglected over the past 20 years. Zimbabwe was offering open heart surgery until 2003, however, due to the deteriorating economic situation, operations ceased only to restart in 2016. Zimbabwe continues to face challenges in the delivery of open heart surgery to children and adults. The major factors are infrastructure problems, human resources, financing for equipment and disposables, training and upgrading the education of the pediatric cardiac team, salaries for healthcare professionals, and political issues in the country. An analysis of the challenges encountered in the continuity of the delivery of the service as well as the examination of the success stories will help to chart a way forward.

## History of Open Heart Surgery in Zimbabwe

Zimbabwe has been a beacon in the medical field with several success stories including performing open heart surgery in the early 1950s (9). The patient was a 15-year-old who had pulmonary valvotomy with surface cooling and recovered well following the surgery. Subsequent to this, there were visiting teams from Loma Linda University (United States) that carried out open heart surgery in the 1970s and 1980s.

Philanthropic missions provide relief to the few that are operated on, however, they are difficult to sustain and local provision of service is ideal. The Loma Linda University sent a core team in 1988 that helped establish a local resident team that carried out several cases of open heart surgery with remarkable success (10, 11). The training was both local and in collaboration with other international cardiac centers resulting in the establishment of a joint team. Together they began the open heart surgery earnestly in 1989 and were able to leave behind a team that successfully continued the efforts. Between 1989 and 1992, they managed to operate 91 cases of open heart surgery with a mortality rate of 8.7%. Alone, the locally assembled team carried out over 400 open heart surgery cases between 1995 and 2003 and operated on a weekly basis. Unfortunately, due to the changes in the country’s political and economic situation, the program folded in 2003.

Efforts were made to resuscitate open heart surgery between 2003 and 2016, which finally came to fruition in February 2016. This was made possible when Medtronic, a private supplier in the medical industry donated a heart-lung (bypass) machine to Parirenyatwa Group of Hospitals in Harare. The University of Zimbabwe, College of Health Sciences had a collaboration with a congenital heart team from the University of Konkuk South Korea (12). The visiting cardiac team led by a Professor in Congenital cardiac surgery, brought full theater sets, consumables, and medication. They performed 11 (mainly pediatric) cases during an 8 day camp, with assistance from the local staff.

The camp built capacity in the local team which continued once the visitors had left, however, they faced teething problems resulting in a limited number of operations. The team from South Korea led by the same Professor visited and performed the surgery again in 2017 and 2018, each time coming with a smaller team as they were weaning the local team (12). There was also a visit by an Italian team, Mission Bambini, in July 2018, and they held a camp for pediatric cardiac surgery. Mission Bambini also brought their own equipment, consumables and medication. The camps by the visiting teams have been very useful in building local capacity and developing the team work required in cardiac surgery. The main emphasis was to leave a local team that is competent to work on its own, hence there was much less material support with the expectation that a sustainable program should be primarily supported locally.

The local team performed several cardiac operations between 2016 and 2018 and these were mainly for rheumatic heart disease. There were sometimes several months between the performance of procedures due to the lack of resources. In the last case, a mitral valve replacement was done in October 2018 and since then the team has been struggling to secure the consumables required.

## Infrastructure, Equipment, and Consumables

The country has one unit, Parirenyatwa Group of Hospitals in Harare, offering open heart surgery. The hospital is the largest in the country and caters to most medical specialties thus there is competition for resources. Parirenyatwa offers cardiac surgery services, however, they are not optimum. There are no private hospitals that offer open heart surgery in Zimbabwe. Bulawayo, the second largest city, and Mutare, the fourth largest city in the country offer echocardiography services, which are performed mainly by pediatricians.

The initial capital cost of the equipment required to run a meaningful program on open heart surgery remains one of the major obstacles. Sophisticated machinery and equipment are essential to carry out these operations (7). There is a need for a cardiac catheterization laboratory (cathlab), a modern heart-lung machine, modern theater trays, and the consumables that are used on day to day basis. The cardiac catheterization laboratory is useful both as a diagnostic and therapeutic tool and is essential in a fully-fledged cardiac unit. There used to be a cathlab at Parirenyatwa which disintegrated from lack of usage. The heart-lung machine is a four-pump head and is out of production. It performs the required function though it’s not ideal, it is also expensive to service. The other equipment such as intra-aortic balloon pump, extracorporeal membrane oxygenator, *trans*-esophageal echocardiography, are not available. This restricts the surgery that can be performed as well as the support services for patients in critical condition. Most of the equipment and consumables that are required for open heart surgery are sourced out of the country and thus require foreign currency. This foreign currency has become a scarce resource due to the economic difficulties facing the country.

Integral to open heart surgery is a Cardiothoracic critical care unit. Almost all patients require admission into the Intensive Care Unit (ICU) or High Dependency Unit (HDU) after cardiac surgery and in the strictest sense, there should be an independent Cardiothoracic ICU and HDU. Unfortunately, the team uses the general ICU which means cardiac beds are not guaranteed as most of the time the units are overflowing. In some cases, there have been special arrangements to secure beds, but it is ideal to have an independent cardiac ICU with at least five ICU and five HDU beds. This would allow the team to provide meaningful service, the proposal being at least three cases a week as this would meet the criteria that are required to keep a single surgeon competent. There has been an increase in the number of ventilators and monitors in ICU/HDU due to the increased demand from the Covid-19 pandemic. A major drawback in ICU is the nursing personnel that is required to man the units, there has been massive decimation of the staff as they leave for greener pastures within and out of the country. The Covid-19 pandemic has resulted in the increased demand for ICU-trained nurses locally, regionally, and internationally and this has worsened the flight of our skilled staff.

Consumables for the bypass machine, valves, rings, and pharmaceutical agents that are used during surgery are mainly sourced out of the country. The main reason the performance of open heart surgery stopped was due to the lack of consumables. There is a need for a dedicated fund for the sustenance of surgical services.

## Health Professionals

**FIGURE 1:**
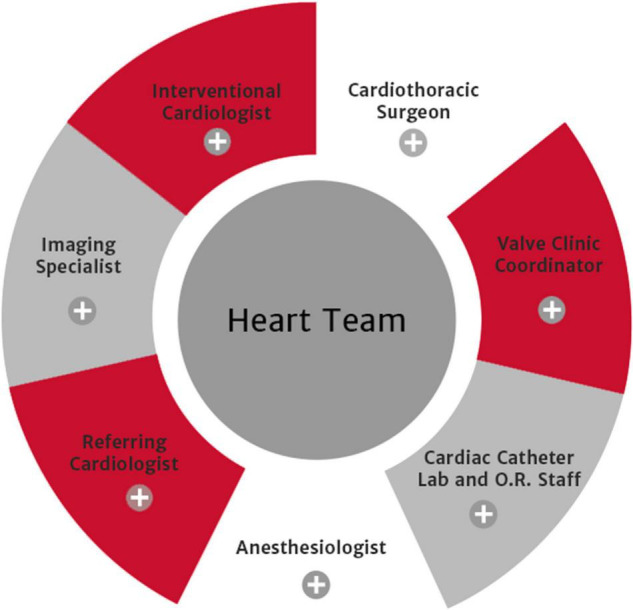
Heart Team from Edwards Lifescience.

The Heart Team Concept is the accepted standard in the major cardiac centers worldwide (13, 14), Figure 1. This is a multi-disciplinary team that manages the patient together with pre-operative and post-operative meetings and audits of the team performance. The following constitute the team: anesthetists, cardiologists, cardiothoracic surgeons, intensivists, nurses (floor, theater, ICU/HDU), perfusionists, and adjunctive staff. Zimbabwe broadly has all the staff required locally available, although they may not be in Government employment.

There is one Cardiothoracic surgeon at Parirenyatwa Hospital at the moment (October 2021) and one who is on attachment in another country. It is encouraging to note that there are at least four registrars in South Africa that are studying for the Fellowship in Cardiothoracic surgery. This demonstrates there is potential to grow the provision of services to cater to the country. It is worthwhile to emphasize that there is a need to make it attractive for the said colleagues to return with their precious skills so that we can grow the specialty. Some of the Doctors are already working on provisions to be employed in South Africa and the greater diaspora as they see that without a functional open heart surgical unit, there are no prospects of applying the skills they have acquired.

The country had one Pediatric Cardiologist from 2009 up until 2021 when a recently qualified colleague returned to the country. The older cardiologist is now in retirement, however, he continues to support the Government Hospitals by offering his services. He has made numerous attempts to train junior colleagues, but unfortunately, they have not been retained in the department.

In terms of the training of doctors instrumental to cardiac surgery, there has been a lot of headway made. The country managed to get its first female pediatric/congenital cardiologist in 2021, she completed her training in South Africa and returned. She is based in Bulawayo the second largest city in the country. In October 2021 two candidates graduated as cardiothoracic surgeons, including again another first female cardiothoracic surgeon. The hope is that they return and serve the nation and that they get the requisite support from all the team players to encourage them to stay. They will form the core of the team that will have the task of ensuring that the unit becomes a center of excellence in the region and the world at large.

The Department of Anesthesia is fairly well staffed and does not have any major problems with the provision for open heart surgery. The anesthetists are also in charge of critical care and thus the post-operative care is smooth as the ones who are responsible for theater take care of the patient.

The heart-lung machine is essential and necessity for cardiac surgery, and it is operated by a perfusionist who needs to be well trained in the operation and running of the machine. Zimbabwe has a single perfusionist who is locally available to run this piece of equipment. The individual is in retirement and has annual renewals of her contract. There are efforts to get two individuals to train in order to address this precarious situation. The long term goal is to embark on a local perfusionist training program once the unit has reached a critical level of operation and staff service.

The nursing staff is one of the most important services in cardiac surgery, they are important in the general ward, in theater, and the critical care unit (ICU and HDU). There is a general shortage of nurses in the country, they easily secure jobs in the diaspora, and thus they leave once they find greener pastures. The main impact of the shortage is in the specialized nurses for theater, ICU/HDU and to a certain extent the renal nurses. The level of staff has a bearing on the number of cases that can be done as there are limited beds in the ICU. There is ongoing training of nurses, however, the rate at which they leave cannot be matched by the new ones coming in.

Supporting services are also a part and parcel of the team for the success of cardiac surgery. The radiological services are required round the clock, personnel shortage results in delays in obtaining postoperative x-rays in ICU and HDU. There is a single portable machine that caters to the hospital and there are major challenges when it breaks down as there are no portable x-rays done at this stage. There are complex cases that may need evaluation using CT-scan or MRI, these facilities are either not working or unavailable at the hospitals.

Laboratory services are essential as well, they allow the constant monitoring of the function of essential organs and the metabolic status of the patient. Some investigations are sublet to private laboratories due to lack of capacity at the local unit. The arterial blood gas machine has frequent breakdowns and also runs out of consumables that are required.

Other departments also play a part in cardiac surgery and have shortages although these do not affect the running of the program as much as those stated above. The pharmacy, physiotherapy, nutrition, and technicians for the cathlab are some of the staff in this case.

The deterioration of the economic conditions has caused disenchantment of the health workers and as a result, there have been industrial actions. There has been significant movement of personnel from Government to private facilities which offer better working conditions. Recruitment by agencies has also resulted in significant emigration to Western countries as health personnel look for brighter prospects. The retainment of staff locally through monetary as well as non-monetary benefits will help stabilize the sector and to ensure growth. Benefits that will make a meaningful impact on the lives of most are a salary that does not depreciate in value, accommodation or provision of land to build, transport, or motor vehicles, medical insurance, and a better working environment that is competitive and up to world standards. If headway can be made in these issues we will be able to retain the critical staff required for the sustenance of cardiac surgical services.

## Politics and the Government

Undoubtedly one of the issues that have had a major bearing on cardiac surgery is the negative political sentiment toward the Zimbabwean Government by the major Western powers. The country has been isolated and frozen out of major financial and political institutions.

Zimbabwe’s political and economic problems that led to the multiple meltdowns commenced in the late 1990s. The main issue was the land resettlement that had remained unresolved since independence and thus there was an invasion of farms. Unfortunately, there was poor organization and the world at large did not support the program. Zimbabwe became a pariah state that had multiple international companies withdrawing their support and services.

There were sanctions were imposed on the country and the Government introduced a raft of measures including prioritizing certain sections of the economy. The health sector suffered greatly as it was dependent on donors and as the donors pulled out their funding the sector was in free fall. This greatly affected the open heart surgery services resulting in the halting of operations between 2003 and 2016.

In 2009 there was a global political agreement between the major political parties and this resulted in political and economic stability in the country. Most medical services improved dramatically and eventually in 2016 open heart surgery was resumed in the country, it was not a smooth matter but at least open heart surgery was done. In 2017 there was a change of Government and there were some monetary policy changes that resulted in the change of currency and most of our suppliers were unable to recover their money from the country. The last case of open heart surgery was carried out in October 2018 when consumables ran out and since then engagements have been made with the Government and other stakeholders to secure support resuscitating the service.

Zimbabwe still continues to face its political challenges, it is now having a positive economic projection which should have a positive bearing on cardiac surgery. It remains the Government policy to ensure health for all its citizens and there is a promise to fund health beyond the 15% of the national budget as advocated in the Abuja declaration.

## Finance

Financing the open heart surgery program has been a major challenge with the sustainability of open heart surgery. There is a need for a major capital input to modernize the unit and a sustained injection of money annually to make it a success. The major benefactor should be the Government of Zimbabwe, the largest employer of health workers and it is in charge of the only hospital in the country that is able to offer open heart surgery at the moment. The government’s sustained support should be complemented by other players in the health industry such as medical insurance companies.

The team made an appeal to Government to assist in modernizing the unit and the Ministry of Health and Child Care (MOHCC) availed USD $3.5 million in 2017 for the refurbishment of the unit. A change in currency in 2017 resulted in the money allocated for the major refurbishments losing value, as a result, the project failed to materialize. Companies that were supplying consumables were also unable to repatriate their money and they thus required cash on delivery which led to the collapse of open heart surgery.

Medical insurance companies cater to less than 10% of the population of Zimbabwe in terms of health coverage, this is a very small part of the population (15). Some patients have been financed in part or in full to have cardiac surgery out of the country when such services were not available in the country. The association of medical insurers locally has not been forthcoming with the estimated total cost of the surgery done out of the country. The insurance companies should make an effort to support the local services. There are cases that can be done locally without the need for cardiopulmonary bypass that are paid for out of the country by the same companies. If these funds are directed toward the local program no doubt they will make a significant difference and reduce the expenditure on foreign currency.

Locally based companies, not in the medical insurance business, can make a very big difference by supporting the program, this can have tax benefits for them. It will also attract more capital for their projects if supporting foreign investors recognize that the country offers world class medical services as they would feel they and their employees are medically covered.

One of the major limitations with surgery was that the majority of the needy patients were unable to afford the cost of surgery. In 2017 the National Oil and Infrastructure Company in Zimbabwe (NOIC) came to the table with a donation of USD $350 000.00 for the patients that needed assistance. This fund went a long way in helping these patients as heart surgery would have remained a pipe dream. There have been other donations from individuals, companies, and non-profit organizations that have supported some of our patients.

Non-governmental organizations, well-wishers and other well-to-do individuals should also be encouraged to come on board. Due to the economic and political changes affecting Zimbabwe for the past two decades, there is now a significant population in the diaspora. This segment of the population has been helping significantly with the remittance of funds, equipment, and consumables supporting the hospitals. The majority of the population would not be able to access such services as they are catastrophic to their meager resources. It is important to encourage those that can contribute what they can to the payments required as this will help. There has been a lot of talk about national health insurance, perhaps if this is achieved it will go a long way toward achieving our goal.

## The Cost of Open Heart Surgery

The overall cost of open heart surgery remains quite steep for the majority of the Zimbabwean population. There is specialized equipment such as the heart-lung machine, repeated x-rays, special medications, blood and blood products, and admission into ICU. Significantly the consumables used on the bypass machine are all sourced out of the country and thus require the scarce foreign currency.

The cost for repair of simple defects like atrial septal defects and ventricular septal defects is between USD $4000 and $6000. The more complex cases and those requiring valve replacements may require up to USD $12000, for example, aortic and mitral valve replacements. These costs are inclusive of the whole hospital stay, consumables, and surgery and they are favorable compared to America and South Africa (7). Costs are similar to those in other African countries (7, 16). However, India offers competitive prices, mainly due to the volume of patients they do this they can negotiate better packages. Unfortunately, due to limited patients, the costs in Zimbabwe remain very high. Perhaps it is time that African countries push to buy their medical consumables in bulk as this would result in a significant reduction in the cost.

## Discussion

It is clear that open heart surgery can be done in Zimbabwe as has been demonstrated by the number of cases that have been done before. The major hurdle is in securing the priorities required for sustainable open heart surgery service delivery. The cost of doing heart surgery has been one of the major obstacles and it results in neglect of the sector. Table 1 shows the number of cases done per year since the resumption in 2016 and is evidence that open heart surgery can be done successfully.

**TABLE 1:** Number of open heart surgery cases per year.

	2016	2017	2018
Children	10	8	16
Adults	15	17	15

The success of the program requires a multi-sectoral approach and a unity of purpose. There is a need for good leadership and perseverance to maintain continuity, this will encourage the younger generation of professionals to remain in the country. Training of personnel locally will then be possible and it has been shown that it is easier to retain locally trained personnel (7). The growth of the unit should also allow the services to be performed in other parts of the country as the service is not commensurate with the population.

Developed countries have the highest rate of performance in open heart surgery and this is closely linked to the per capita gross domestic product (7). Countries in similar situations as Zimbabwe have managed to perform open heart surgery despite their economic, political, and social setbacks (7, 16–20). Zimbabwe should emulate these countries in order to have a locally sustainable program. A comparison of the GDP of some countries that are offering OHS shows that Zimbabwe is better off than other countries that are offering cardiac surgery (Table 2) (21).

**TABLE 2:** Comparison of GDPs in selected countries.

Country	GDP in US$ (2020)
Zimbabwe	1,214.5
Angola	1,776
Bangladesh	1.961.6
India	1,927.7
Kenya	1,878.6
Uganda	822.0
Tanzania	1.076.5

There are continued engagements with the MOHCC in an effort to resuscitate open heart surgery. Multiple meetings have been held and the consumables, equipment, and repairs that are required are listed and budgeted for. The finalization of the budget allocation is still pending hence operations are still to recommence. The major renovations and upgrading of the unit as had been allocated funding previously are all on the new plans that have been presented to the MOHCC.

The team is in communication with the administration at Parirenyatwa Hospital on the plan to resume services. To allow the unit to function smoothly there is a need for a separate account for cardiac surgery, with particular emphasis on payment at least for the consumables in foreign currency. There is a need to regularly replenish the consumables so as to avoid stock outs. To assist the needy there can be a priority fee that is charged to private patients.

The hospital is also urged to market itself as the only unit in the country that can perform open heart surgery. Medical insurance companies have covered some of their members who have had cardiac surgery locally, and marketing to them continues to be a priority. Appealing to the corporate world to practice corporate social responsibility will make a difference as seen by the assistance received from NOIC. Taxation may be exempted for such companies for their noble cause.

The local team continues to partner with the team from South Korea and will continue to strengthen this relationship. Not only have they come to assist set up open heart surgery, but they have also taken some of the local staff to work in their units. They accepted a team comprising of an anesthetist, surgeon, and nurses (ICU and theater) who familiarized themselves with a modern set up of a fully functional cardiac unit. The team from Mission Bambini is also a good partner, they took one of our anesthetists who went to Italy and did a Masters in Cardiac anesthesia. There are other units that are enquiring with a view of assisting the unit, and hopefully, when fully functional it will be a center of excellence in the near future.

There is work on coming up with a database of the patients that need cardiac surgery. It is easier to make a case when appealing for funding when the disease burden is known. There is a paucity of literature on cardiac disease from African sources, we aim to contribute to the papers already locally produced (6, 9–12, 22).

Patients have made plans of their own to get operated out of the country and the main destinations are South Africa and India. South Africa is a neighboring country that hosts a large Zimbabwean population, this makes it attractive and easier for most patients. It has a well-developed health system with many hospitals that offer open heart surgery both private and public. The private facilities in South Africa are much more expensive than those in India. The cost of surgery in India is much less hence more patients go there despite it being much further away. Medical tourism is not without its drawbacks it usually requires one to have a Visa which may result in costly delays. The follow-up care for patients that have complications and those that require to follow-up surgery is not optimal. There are also language barriers where most people use their local language. Local regulatory authorities have no control over practitioners in other countries thus recourse for any complaints or malpractice is difficult to obtain. It is difficult and expensive to repatriate bodies if the surgery does not go well, especially without a regular support system. The SARS COV2 outbreak has also resulted in major disruptions to travel therefore even those with the means are unable to access services.

The cost of cardiac surgery is quite significant for the majority of the patients, especially if one looks at it as a once-off payment. There is a tendency to overlook the cost of repeated medications, and the repeated and sometimes lengthy admissions that patients have to endure. It is costly socially as well as parents have to be with children when they are not well or hospitalized. There is the neglect of other children, work, and social commitments. When the patients succumb to heart diseases the emotional cost cannot be quantified.

The patients themselves have not been sitting back and waiting, they have their patient advocacy groups like Brave Little Hearts and ZimHearts. Through these groups the patients are able to meet and exchange ideas on their conditions, some can be directed to the relevant specialists. Some patients are assisted with funding for medication and surgery and most of all it assists with raising the awareness in society about heart problems. The organization Brave Little Hearts has championed the advocacy and awareness campaign for congenital heart diseases. Through their efforts, they helped equip a pediatric cardiac ICU/HDU unit at Mpilo Hospital in Bulawayo that was officially opened on World Heart Day 2021.

## Recommendations on the Way Forward

1. Continued engagement with the Government through MOHCC to provide capital for consumables for the immediate resumption of open heart surgery. Sustained support of the program and honoring the capital investment in the unit.
2. Incentives to retain local personnel by monetary and non-monetary rewards. Training of the staff to meet the local demand and to stay abreast with world standards.
3. Opening a special account for open heart surgery that allows timely replenishment of resources.
4. An independent Cardiothoracic ICU and HDU with at least five beds for each unit, to allow smooth operations.
5. The continued association and new partnership with established cardiac centers to exchange ideas and allow for fellowships for training.
6. Encouragement of support by the local major industry as part of social corporate responsibility.

## Conclusion

Zimbabwe has the potential to provide sustainable open heart surgery services which are sorely missed by cardiac patients. The cost of cardiac surgery remains the main prohibitive factor, especially as the country grapples with economic difficulties. This should not be a deterrent as there are other countries that have been in similar situations or worse off that have prevailed. There is a need for strong, persuasive, and perseverance in the advocates for cardiac surgery. The Government should be the main benefactor and should be encouraged to give unwavering support to the team. All players that can assist in the goal should be encouraged to come on board and get the ball rolling.

## Author Contributions

All authors listed have made a substantial, direct, and intellectual contribution to the work, and approved it for publication.

## Conflict of Interest

The authors declare that the research was conducted in the absence of any commercial or financial relationships that could be construed as a potential conflict of interest.

## Publisher’s Note

All claims expressed in this article are solely those of the authors and do not necessarily represent those of their affiliated organizations, or those of the publisher, the editors and the reviewers. Any product that may be evaluated in this article, or claim that may be made by its manufacturer, is not guaranteed or endorsed by the publisher.
